# Reliability and Time Efficiency of Digital vs. Analog Bite Registration Technique for the Manufacture of Full-Arch Fixed Implant Prostheses

**DOI:** 10.3390/jcm11102882

**Published:** 2022-05-19

**Authors:** Philippe Nuytens, Rani D’haese, Stefan Vandeweghe

**Affiliations:** 1 Private Practice for Restorative Dentistry, Het Tandatelier, 8870 Izegem, Belgium; philippe.nuytens3@gmail.com; 2 Department of Reconstructive Dentistry, Dental School, Faculty of Medicine and Health Sciences, Ghent University, 9000 Ghent, Belgium; rani.dhaese@ugent.be

**Keywords:** bite registration, implants, full-arch, complete arch, digital impression, intraoral scan, digital workflow, bite pillar, dual function scan body

## Abstract

Objective: Information about full-digital protocols for bite registration with intraoral scanners on multiple implants in the edentulous jaw is scarce. The purpose of this comparative in vivo study was to investigate the reliability and time efficiency of a novel full-digital bite registration technique for the manufacture of full-arch maxillary fixed implant prostheses. Material and methods: In ten patients, a full-arch maxillary fixed implant prosthesis was manufactured on multi-unit abutment level through an analog prosthetic workflow. The bite registration was performed with use of a screw-retained polymethyl methacrylate (PMMA) verification jig with detachable wax rim. To articulate the definitive edentulous maxillary implant cast in centric relation at the appropriate occlusal vertical dimension (OVD) to the mandibular antagonist cast, a type II articulator (Artex, Amann Girrbach) was used. Three to six months later, a full-digital bite registration was performed with use of dual-function scan bodies and bilateral connected bite pillars. The bite pillars screwed into the scan bodies were used to adjust and articulate the edentulous maxillary implant arch to the mandibular antagonist arch at the defined OVD. Treatment time for analog and digital bite registration technique was measured in each patient. The reliability of the digital bite registration technique was evaluated by 3D comparison of two sets of stereo lithographic (STL) files obtained from each patient. The three-dimensional deviation was defined along the X-, Y- and *Z*-axes (Geomagic Control X, 3D Systems Inc., Rock Hill, SC, USA). Results: The treatment time for digital bite registration using dual-function scan bodies and bite pillars was significantly shorter than analog bite registration with verification jig and wax rim (60.30%, SD 5.72%). Minor differences between the two techniques were observed with a linear deviation range of 1115 µm (SD 668 µm) overall, 46.2 µm (SD 731.3 µm) along the *X*-axis, −200.3 µm (SD 744.3 µm) along the *Y*-axis and 67.1 µm (SD 752.2 µm) along the *Z*-axis. Bilateral balanced contacts were registered in all patients during full-digital bite registration. Conclusions: The novel digital bite registration technique with dual-function scan bodies and bite pillars allows for a full-digital workflow for full-arch implant supported restorations. The digital bite workflow was 60% faster, and the overall deviation was around 1 mm, which can be considered clinically acceptable.

## 1. Introduction

Mounting an edentulous cast at the appropriate occlusal vertical dimension (OVD) and in centric relation (CR) is a crucial step in the rehabilitation of edentulous patients [1,2], certainly for the manufacture of a full-arch fixed implant prosthesis [3]. The correct OVD is determined by the rest position of the mandibular arch [4,5,6,7] and is defined by the point at which prosthetic tooth contact is made along the arc in centric occlusion (CO) or retruded contact position (RCP) [8]. The retruded contact position (RCP) was first introduced in 1952 in Posselt’s sagittal envelope of mandibular border movements’ as a relatively reproducible maxillomandibular relationship and plays a key role for mounting casts on an articulator [9,10,11,12].

Because occlusion has a biological adaptability and is not constant, we may speak of a range of CO positions acceptable for prosthetic rehabilitation. This freedom in centric was first described in 1969 by C. H. Schuyler as a flat area (500–1000 µm) created between centric relation and centric occlusion positions on the occlusal surfaces, from hinge position to habitual intercuspal position without any change in OVD [13]. To obtain a consistent CO registration, some authors recommended using mandibular guidance from the operator, chin point guidance, bi-manual manipulation or the use of an anterior jig [8,12].

A common registration technique for mounting the edentulous implant cast for a full-arch fixed implant prosthesis is the use of a screw-retained wax rim. This technique ensures a transfer of the implant cast at the correct vertical dimension (OVD) in centric relation (CR) to the articulator. Alternatively, a detachable wax rim supported by a verification jig can be made. This simplifies the procedure and avoids the entrapment of wax in the screw-access holes. In addition, the verification jig serves as a verification of the accuracy of the master cast. Other options for bite registration involve the use of a duplicated denture with acrylic resin as a custom impression tray, or even a duplicated denture with impression material [14,15,16,17]. Nevertheless, bite registration materials and methods should be selected carefully for implant-supported fixed prostheses [18].

The dynamic registration of Gothic arch tracing was first described in 1947 by Aprile and Salzar [19]. This method was found to be more technique sensitive and required more chair-side time both for the dentist and the patient. There was also a greater risk of incorporating errors due to mishandling of the device and fatigue of the jaw muscles from repeated efforts to guide the mandibular movements [20]. In case of an implant-supported prosthesis, healing abutments can be used to stabilize a record base for accurate Gothic arch tracing [21]. However, the use of auxiliary instruments is a drawback for inexperienced dentists to perform an accurate bite registration [20].

In digital workflow, the registration of the correct occlusion and manufacture of the prosthesis without physical casts and wax rim is difficult [22]. A full-digital workflow has been described for partial edentulous cases [23] but is still challenging for full edentulous cases.

For dentate cases, full-digital techniques for mounting a full-arch intraoral scan on a virtual articulator were described in the literature in several clinical case reports [15,24,25,26]. Hong et al. describes a technique to determine the sagittal condylar inclination (SCI) using CBCT data and intraoral scan data of the protrusive interocclusal position [27]. However, these techniques require the presence of teeth for superimposing.

Some authors suggest scanning the provisional restoration to superimpose with the scan bodies in the intraoral scan [17,28,29]. This requires, however, the presence of a provisional restoration that was made in a conventional way [18,30]. Another approach is to section the anatomical-shaped surgical guide and use it as a quadrant support while scanning the scan bodies and jaw relation in the contralateral quadrant [31]. Some authors have proposed the use of a custom scanning device, which is a printed or milled copy of the patient’s denture, which contains perforations at the implant locations. This allows the scan bodies to perforate and be scanned together with the custom device. Since most of the outline of this custom scanning device is identical to the complete denture, it provides a stable occlusion and correct OVD to register the maxillomandibular relationship. Although these concepts provide a solution to perform the digital workflow in edentulous arches, physical auxiliary instruments are still necessary [17,32]. A more invasive option is to place additional reference pins or mini-implants in the bone, which serve as markers for superimposing different scans [33,34,35]. Although these techniques have been successfully used in clinical studies, they require additional treatment steps or appliances and are therefore not always applicable in daily practice.

The aim of this study was to validate a novel technique for full-digital bite registration with a dual function scan body system providing dual functionality on multiple implants in the edentulous jaw. The first null hypothesis is that there is no difference between the digital and analogue bite registration in terms of treatment time. The second null hypothesis is that the CR position could be achieved at a pre-defined OVD within a clinically acceptable range of 1 mm in all axes.

## 2. Materials and Methods

### 2.1. Design of the Study

In ten patients, a full-arch maxillary fixed implant prosthesis was manufactured on multi-unit abutment level through an analog prosthetic workflow. In seven patients, the prosthesis was manufactured on 6 maxillary implants. In the other patients, 4, 5 and 7 maxillary implants were used to support a full-arch fixed implant prosthesis.

**Figure 1:**
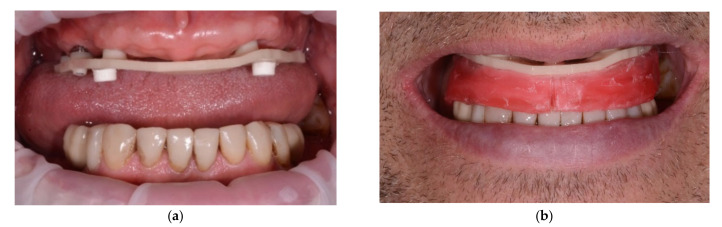
The analog bite registration was performed with use of a screw retained verification jig and a detachable wax rim: (**a**) screw retained PMMA (polymethyl methacrylate) verification jig on multi-unit abutment level with bilateral attachments (LEGO SYSTEM A/S, Billund, Denmark); (**b**) adjusted wax rim in CO position attached to the PMMA verification jig.

In all patients, multi-unit abutments (Medentika, Hügelsheim, Germany) were installed at a torque value of 15 Ncm prior to the full-arch impression. The bite registration was performed with use of a screw retained verification jig and a detachable wax rim (Figure 1a,b). A passive fit was observed in all patients.

Treatment time for the analog bite registration technique was measured for each patient, starting from mounting the verification jig onto the multi-unit abutment level and ending with the manually mounted physical casts ready to send to the dental laboratory. To articulate the definitive edentulous maxillary implant cast at the appropriate occlusal vertical dimension (OVD) in centric relation to the mandibular antagonist cast, a type II articulator (Artex, Amann Girrbach) was used in the dental laboratory. A stopwatch (CASIO HS-80TW-1EF, CASIO COMPUTER CO., LTD., Shibuya-ku, Tokyo, Japan) was used to record the clinical time required for the two bite registration methods. Time was recorded by an independent investigator who was informed about the study protocol before study initiation.

**Figure 2:**
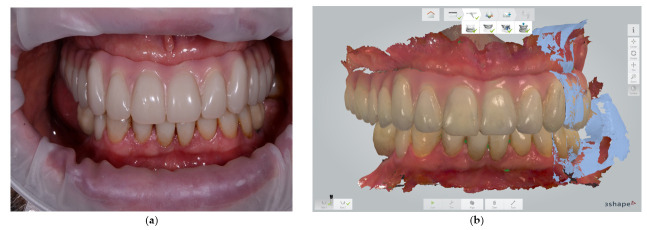
(**a**) Intraoral clinical view 4 months after installation of the maxillary full-arch fixed implant prosthesis on multi-unit abutment level. (**b**) The maxillary scan with the full-arch fixed implant prosthesis was saved in pre-preparation mode (3Shape Dental Desktop 1.7.9.1, 3Shape, Copenhagen, Denmark).

**Figure 3:**
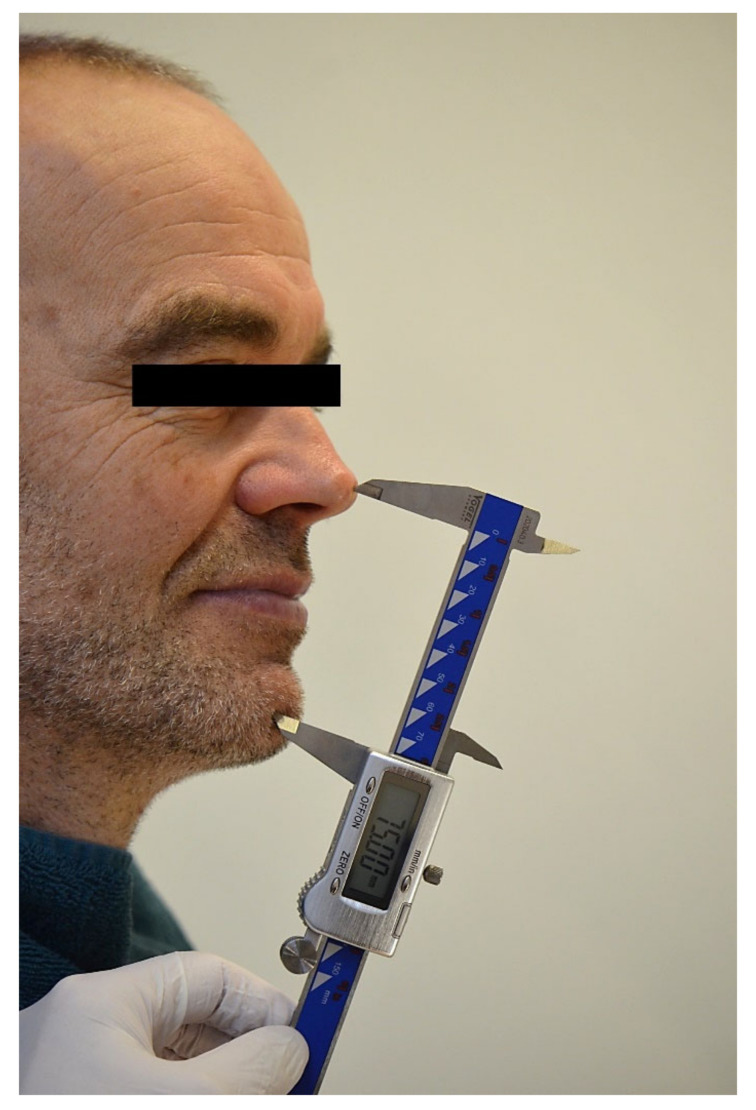
The patient was asked to close with the full-arch fixed implant prosthesis in place, and the distance between a pen dot on the patient’s nose and chin was measured with a digital caliper.

Three to six months later, an intraoral scan of the maxillary arch with the full-arch fixed implant prosthesis in place was taken and saved in pre-preparation mode (3Shape Dental Desktop 1.7.9.1, 3Shape, Copenhagen, Denmark) (Figure 2a,b). The patient was asked to close, and the distance between a pen dot on the patient’s nose and chin was measured with a digital caliper (Figure 3).

**Figure 4:**
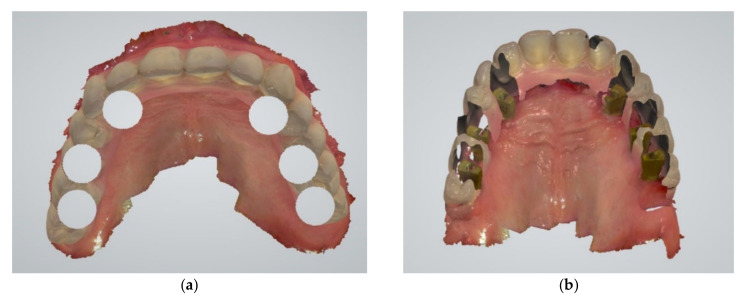
Subsequent to the intraoral scan of the maxillary arch with the full-arch fixed implant prosthesis: (**a**) a cut-out diameter of 6 mm was set for each implant position; (**b**) scan parts of dual-function scan bodies were installed and the maxillary scan was finalized by scanning the cut-out regions.

**Figure 5:**
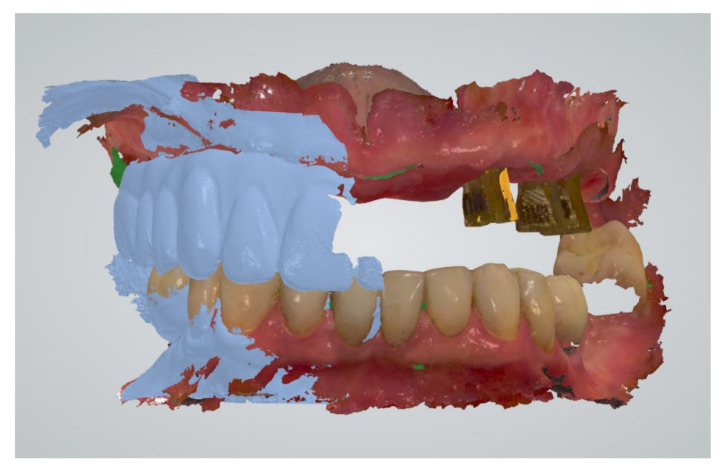
The bite registration was performed with two buccal bite-scans tracing the full-arch maxillary fixed implant prosthesis and the mandibular antagonist arch (3Shape Dental Desktop 1.7.9.1, 3Shape, Copenhagen, Denmark). Consequently, the scan body positions are linked to the original maxillomandibular relationship as determined by the analog bite registration.

For each implant position in the full-arch maxillary fixed implant prosthesis, a cut-out diameter of 6 mm was set in the intraoral scan. Next, the mandibular arch was scanned, and the bite was registered by taking two buccal scans, left and right, while the patient closed with the fixed prosthesis still in place. After disconnecting the full-arch fixed implant prosthesis, the dual-function scan bodies were installed. The maxillary scan was finalized by scanning the cut-out regions (Figure 4a,b) and registering the positions of the scan bodies. The maxillary scans were now aligned with the bite-scan, thereby positioning the maxilla with scan bodies in the original maxillomandibular relationship as determined by the analog bite registration, as used to create the current prosthesis (Figure 5).

**Figure 6:**
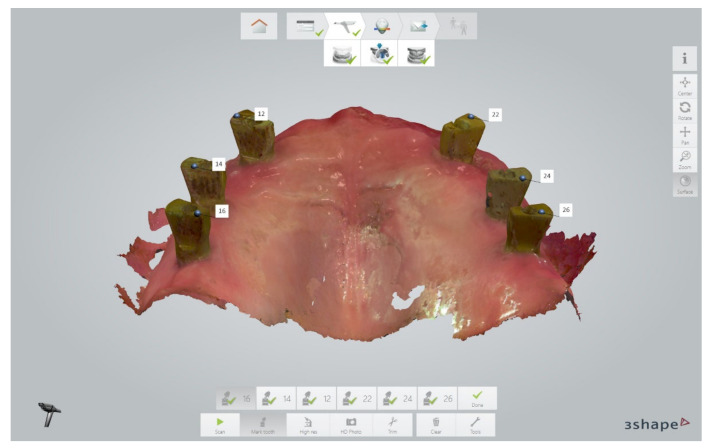
Intraoral scan of the maxillary arch with the scan parts of the dual-function scan bodies during the second scan session (3Shape Dental Desktop 1.7.9.1, 3Shape, Copenhagen, Denmark).

In a second session during the same visit, a new case was created in the intraoral scanner software (3Shape Dental Desktop 1.7.9.1, 3Shape, Copenhagen, Denmark), and the maxillary arch was scanned with the scan parts of the dual-function scan bodies on multi-unit abutment level (Figure 6).

**Figure 7:**
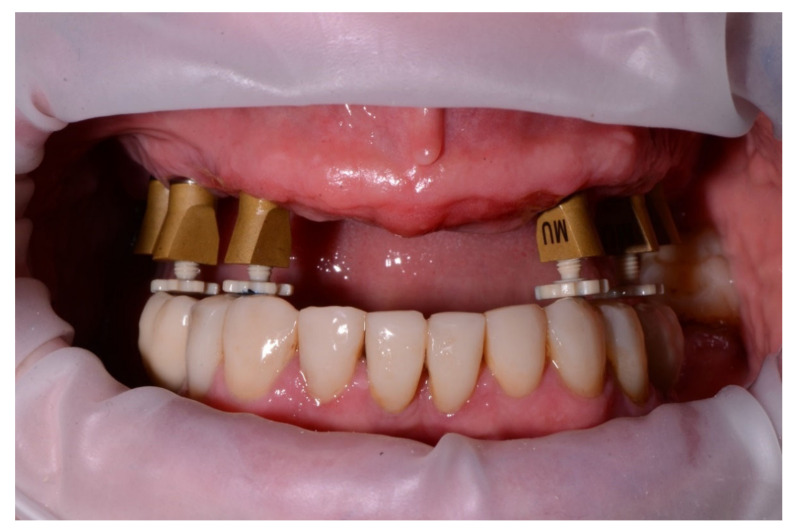
Bite pillars inserted into the screw holes of the scan parts of the dual-function scan bodies mounted on multi-unit abutment level, adjusted at the desired occlusal vertical dimension (OVD).

**Figure 8:**
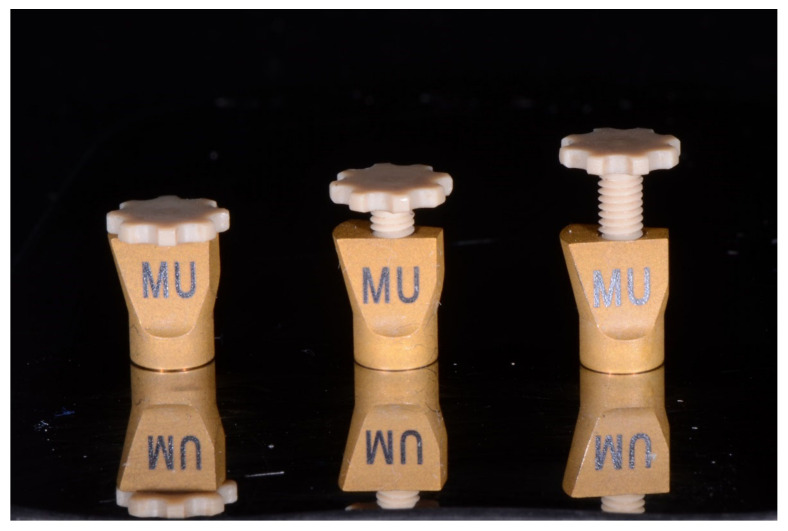
Dual-function scan bodies on multi-unit abutment level (MU). The screw channel of the scan body contains a screw-thread to attach the bite pillar and to adjust it to the correct vertical height.

**Figure 9:**
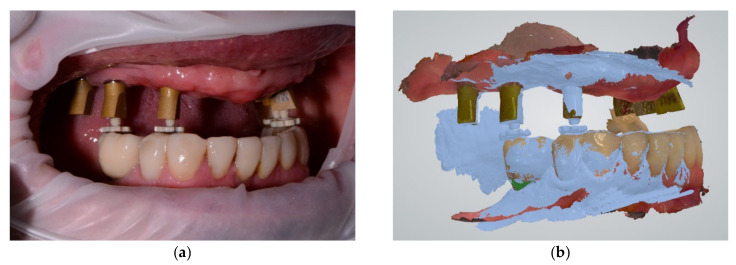
(**a**) Right lateral side with a stable contact on four bite pillars screwed into the scan parts of the dual-function scan bodies at the desired OVD. (**b**) View on screen after digital bite registration at the right lateral side (3Shape Dental Desktop 1.7.9.1, 3Shape, Copenhagen, Denmark).

The digital bite registration started with the insertion of bite pillars into the screw holes of the scan parts of the dual-function scan bodies (Figure 7). Bite pillar prototypes of 3 different lengths were fabricated in PEEK material and were available for this study. These bite pillar prototypes allow for a maximal extension up to 7 mm from the upper surface of the scan part (Figure 8). The defined OVD, set by the extra oral marks, was obtained by adjusting the bite pillars at the desired position in contact with the opposing arch. Two lateral bite-scans were taken to complete the digital bite registration (Figure 9a,b).

The treatment time for the digital bite registration technique was recorded from the installation of the dual-function scan bodies at a torque value of 10 Ncm, installing/adjusting the bite pillars and the registration of 2 lateral bite-scans. The treatment time ended when the digital casts were aligned in the intraoral scanner software and ready to be sent to the dental laboratory.

### 2.2. Data Definition

**Figure 10:**
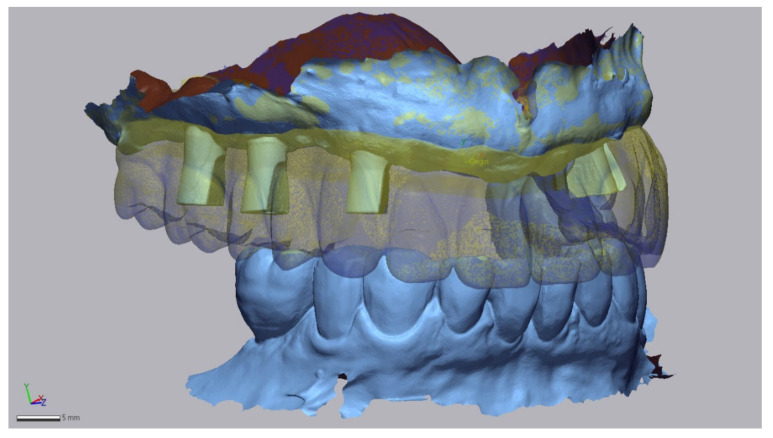
STL-set T1: Scan data of the analog bite registration. A digital impression of the maxillary arch with the established full-arch fixed implant prosthesis after 4 months in situ (transparently yellow) combined with a digital impression of the maxillary dual-function scan bodies, a digital mandibular antagonist impression and two buccal bite-scans (Geomagic Control X, 3D Systems Inc., Rock Hill, SC, USA).

For each patient, 2 STL-sets were collected. The first STL-set (T1) is the scan data based on the analog bite registration and the current prosthesis. It contains an intraoral scan of the maxillary arch with the established full-arch fixed implant prosthesis and the mandibular antagonist at the maxillomandibular relationship as determined by the analog bite registration linked with an intraoral scan of the maxillary dual-function scan bodies (Figure 10).

**Figure 11:**
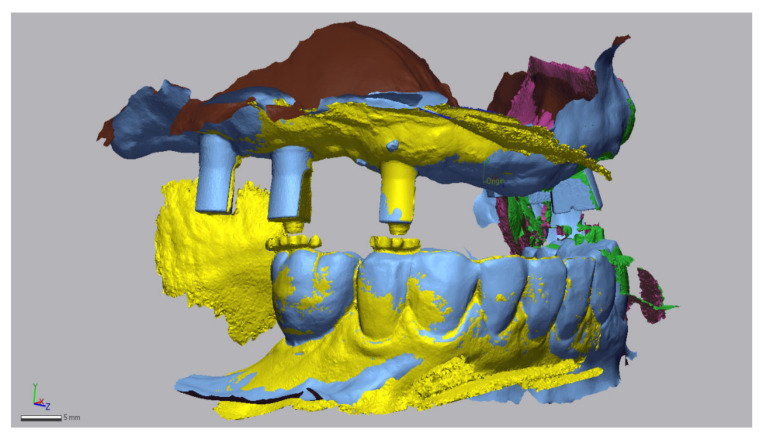
STL-set T2: Scan data of the digital bite registration. A digital impression of the maxillary arch with dual-function scan bodies, a digital mandibular antagonist impression and two bilateral buccal bite-scans with adjusted bite pillars inserted into the screw holes of the dual-function scan bodies (Geomagic Control X, 3D Systems Inc., Rock Hill, SC, USA).

The second STL-set (T2) is the scan data of the digital bite registration and contains an intraoral scan of the maxillary arch with only the dual-function scan bodies, the mandibular antagonist and two buccal bite-scans (left and right) with the adjusted bite pillars at the appropriate OVD (Figure 11).

### 2.3. Linear Measurements

A “best-fit” algorithm based on the mandibular arch was performed (Geomagic Control X, 3D Systems Inc., Rock Hill, SC, USA) to align both datasets. To compare the results of data sets T1 and T2, the X-, Y- and *Z*-axes were equated to enable future interpretation and analysis.

Occlusal plane was defined as X*Y*-axis, with the origin between tooth 41 and 31. To ensure that the direction on the *X*-axis was the same within the first and second quadrant, the absolute value of the *X*-axis was used for the measurements.

The deviation along the *X*-axis represented a medio-lateral movement between the two CO positions. A positive X-value means a more lateral position of the digital bite registration in comparison to the analog bite registration.

Deviation along the *Y*-axis represented a dorso-ventral movement between the two CO positions. A positive Y-value means a more dorsal position of the digital bite registration in comparison to the analog bite registration.

Deviation along the *Z*-axis represented a caudo-cranial movement between the two CO positions. A positive Z-value means a more cranial position of the digital bite registration in comparison to the analog bite registration.

**Figure 12:**
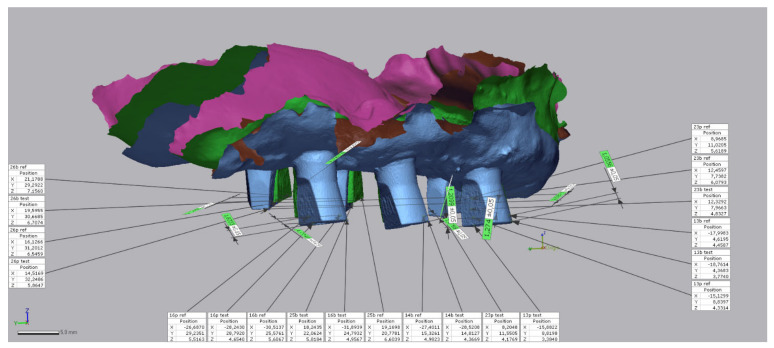
For each patient, 10 analytical points were added to different scan body positions in the maxillary arch. Numerical identification of each analytical point was performed along the X-, Y- and *Z*-axes (Geomagic Control X, 3D Systems Inc., Rock Hill, SC, USA).

For each patient, 10 analytical points were determined on the scan bodies in the intraoral maxillary scan. First, all mesio-buccal corners were determined, followed by the mesio-lingual corners and (in one patient with four maxillary bone level implants) the disto-buccal corners. Numerical identification of each analytical point was performed along the X-, Y- and *Z*-axes (Figure 12), and the overall 3D deviation was calculated for each patient. In addition, the absolute deviation was recorded on all axes to determine the true linear distortion.

### 2.4. Statistical Analyses

Normality was checked with QQ plots, histograms, boxplots and the Shapiro–Wilk test. The Shapiro–Wilk test was not significant for both the linear measurements and the treatment time. One sample *t* test was used to compare the linear measurements. All statistical analyses were performed using SPSS 27, with the level of significance set at *p* < 0.05. Based on a mean difference of 1mm, the sample size was 10 to achieve a power of 80%.

## 3. Results

**Figure 13:**
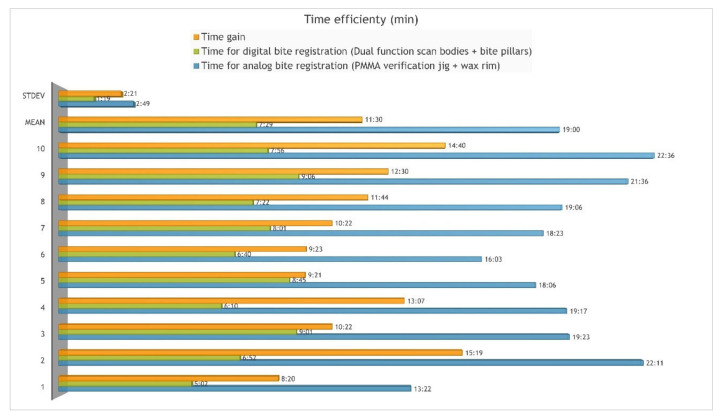
Time efficiency of digital vs. analog bite registration technique.

The mean treatment time for the analog bite registration technique was 19:00 min (SD 2:49 min; range 13:22–22:36 min) and 7:29 min (SD 1:19 min; range 5:02–9:06 min) for the digital registration with dual-function scan bodies and bite pillars. The mean time gain was 11:30 min (SD 2:21 min; range 8:20–15:19 min) or 60.36% (SD 5.58%). The difference is statistically significant (*p* < 0.001) (Figure 13).

Bilateral balanced CO positions were registered in all patients during digital bite registration on at least one bite pillar per quadrant. The mean percentage of scan bodies establishing a stable contact with the antagonistic dentition was 69% (range 50–100%) per patient.

The mean overall 3D deviation between the analog and digital technique was 1115 µm (SD 668 µm, range 150–3160 µm). The mean linear deviation was 46 µm (SD 731 µm, range −2271–2135 µm) along the *X*-axis, −200 µm (SD 744 µm, range −2361–1376 µm) along the *Y*-axis and 67 µm (SD 752 µm, range −1660–1270 µm) along the *Z*-axis (Table 1). Only the deviation along the *Y*-axis was significant (*p* = 0.008).

**Figure 14:**
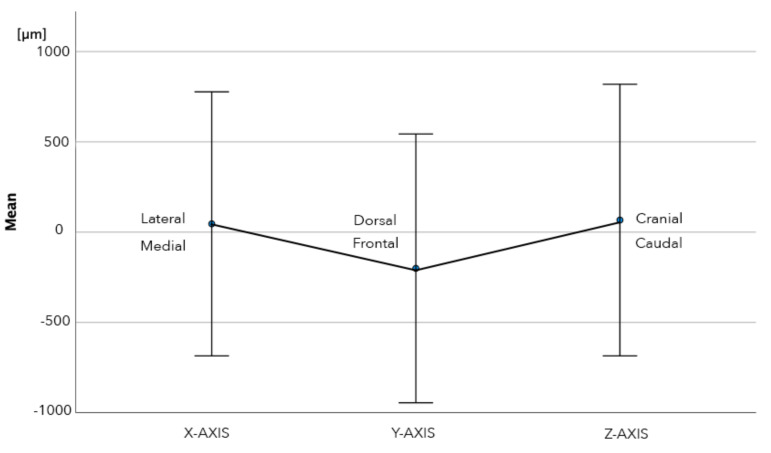
Linear deviation along X, Y and *Y*-axis between analog and digital bite registration technique. Mean and error bars are shown, with the error bars representing 1 SD.

The absolute linear deviation was 475 µm (SD 556 µm, range 10–2270 µm) along the *X*-axis, 611 µm (SD 467 µm, range 10–2360 µm) along the *Y*-axis and 633 µm (SD 407 µm, range 10–1660 µm) along the *Z*-axis (Table 1). These deviations were statistically significant along the *X*-axis (*p* < 0.001), *Y*-axis (*p* < 0.001) and *Z*-axis (*p* < 0.001). The mean absolute 3D deviation in the first quadrant was 1075 µm (SD 692 range 150–3160 µm) and in the second quadrant 1155 µm (SD 647, range 210–2700 µm), which was not statistically significant different (*p* = 0.594) (Table 1) (Figure 14). Both intra-observer (*p* = 0.386, ICC = 0.998) and inter-observer (*p* = 0.114, ICC = 0.945) reliability were high.

## 4. Discussion

The first null hypothesis of this study was rejected (*p* < 0.001), as the time to perform a digital bite registration with the novel technique was significantly shorter (60%) than the conventional analog bite registration. The second null hypothesis was also rejected, as the CR position could be achieved at a pre-defined OVD within a clinically acceptable range of 1 mm in all axes.

Although digital impressions have been proven to be as accurate as conventional impressions for tooth-supported and implant-supported fixed partial prostheses, full-arch implant-supported reconstructions are still challenging to manufacture through a digital workflow. The lack of reference points, due to the missing dentition and ridge resorption, affects the accuracy of the scanning procedure [36]. Recent research has also demonstrated that full-arch digital implant impressions can be made with sufficient accuracy to achieve a passive fit [37,38,39,40,41,42,43]. However, the bite registration still requires physical auxiliary tools, such as a bite rim or tooth guide.

The technique with the bite pillars simplifies the bite registration and alignment of the edentulous arch with scan bodies to the opposing jaw. Apart from the simplified digital registration, this approach also saves time by registering the bite in the same visit as the impression. Since the bite pillars are directly mounted onto the fixated scan bodies, no time is lost during the intraoral scanning procedure to attach or detach various components. The overall treatment time was reduced by more than 50%, resulting in a time gain of 11.30 min. The patients were treated by a clinician who has experience with both bite registration methods. One can expect a learning curve to adapt the digital protocol, which might require more time in the beginning.

Although the elimination of a physical cast and articulator will reduce shipping time and costs to the dental laboratory, this advantage was not included in this comparative study.

Recording the jaw relation is a fundamental and crucial step to provide a well-functioning restoration. An accurate registration minimizes the need for intraoral adjustments and can therefore reduce treatment time and costs. The novel digital bite registration technique is a static registration of the jaw relation, directly captured by an intraoral scanner, and may therefore result in a more anterior registration of the CO position compared to dynamic Gothic arch tracing. This was also observed in our study, where we noticed a significant anterior shift of 200 µm in the jaw relation.

The absolute 3D deviation was 475 µm, which is in line with the findings by Wong et al. [44], who reported distortions around 500 µm in the bite registration of three different intraoral scanners. Fortunately, because of the biological adaptability of the patient’s occlusion, a range of CO positions are acceptable for prosthetic rehabilitation. According to Schuyler et al. [13], the centric freedom is a flat area ranging from 500–1000 µm, arising between centric relationship and centric occlusion positions on the occlusal surfaces, from hinge position to habituated intercuspal position without any change in the OVD. The majority of the measured deviations between the analog and digital registration were within or even below this threshold. Therefore, the digital bite registration using the bite pillars can be considered accurate, despite some deviation, which will have little to no impact on oral functioning. Nevertheless, it has been shown that a total 3D deviation of 1 mm will have limited to no clinical impact on the outcome of the rehabilitation [11,12,13,14]. Small distortions may also be compensated during the CAD design stage or by correcting the occlusion of the restoration during try-in [44].

Intra-oral scanners demonstrate a median deviation between 28–91 µm for full-arch implant scans, depending on the scanning system [45]. Although this deviation also affects the accuracy of bite registration, the clinical impact will be less compared to the influence on the fit and marginal adaptation of the final prosthesis. Intraoral scanners register the static relationship of the maxillary and mandibular jaw with a similar or even better accuracy than the conventional physical interocclusal record [46,47,48,49,50]. Ren et al. found a mean distortion of 280 µm in occlusal relation for digital impressions of partially edentulous casts [51]. In comparison, Eriksson et al. reported a distortion varying from 170 to 2650 µm after mounting the gypsum casts the conventional way [52]. However, similar to the conventional bite registration, the clinician’s experience with the IOS system is critical for the accuracy when recording the CR position [50].

Edher et al. [53] observed a tilting effect of the complete-arch scan toward the site of the interocclusal registration scan. For quadrant scans, they found a higher sensitivity when capturing the CR position. In addition, Gintaute et al. [54] reported a lower precision of the bite registration of full-arch scans when the posterior occlusal relation was scanned in comparison to the anterior relation. In the current study, a digital bite-scan was taken on both sides of the jaws. Since there was no significant difference in the deviation between the first and second quadrant, one can assume that the alignment of the maxillary and mandibular scans with the bite-scan was correct and not tilted to one side.

An additional argument for the observed deviations may be related to the determination of the OVD using a digital caliper. This is not an accurate measurement method and will affect the distortion in all directions, although this has limited impact on the digital bite registration method.

The accuracy of intraoral scanning is influenced by various factors, such as operator experience [55,56,57], scanning device, scanning range, inter-implant distance and the scan body design [29,58]. The height of the basal scan part of the dual function scan body is a crucial factor in the digital bite registration process, since its amount of visibility will affect the accuracy of the implant position registration [59,60]. The initial prototypes of the dual-function scan bodies were designed and manufactured on implant level, under supervision of the inventor (P.N.) and often resulted in a deep submucosal placement of the scan body, with little visibility on the intraoral scan to perform a correct alignment in the CAD-software. Therefore, the use of multi-unit abutments is advocated, which will compensate for variations in the mucosal thickness and corrects the implant angulation when necessary.

Conversely, the suggested protocol also requires mouth closure at the correct OVD without interference of the scan bodies. Therefore, the length of the scan bodies should be limited. The height of the basal scan part of the scan body (=7.5 mm) was based on personal feasibility tests by the inventor (P.N.), who measured the length of 1600 screw-access channels in partial- and full-arch implant restorations from March 2015 to October 2017 [61]. In none of the reported cases in this comparative in vivo study, this led to an interference with the opposing arch, nor did it hamper the alignment in the intraoral scanner software (3Shape Dental Desktop 1.7.9.1, 3Shape, Copenhagen, Denmark).

The use of the bite pillars and dual-function scan bodies allows for a full-digital workflow, in which the restoration is designed and fabricated in a monolithic approach without the need for a 3D-printed cast. This will further simplify the treatment and reduce the costs.

**Figure 15:**
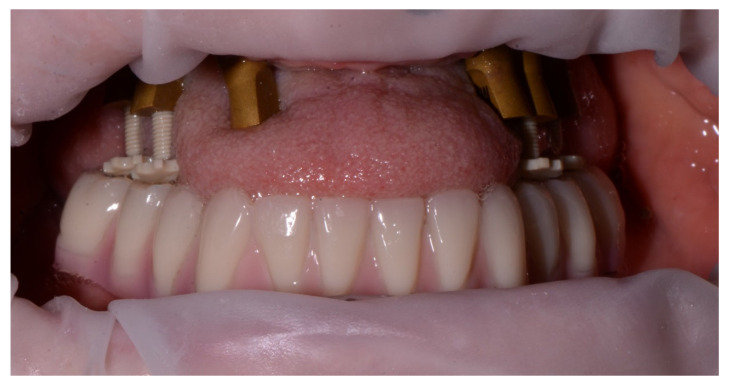
Female patient of 61 years old with severe crestal bone loss in the maxillary arch and voluminous tongue interposition. Long bite pillars are recommended to set the maxillary arch in the appropriate OVD to the mandibular antagonist arch.

**Figure 16:**
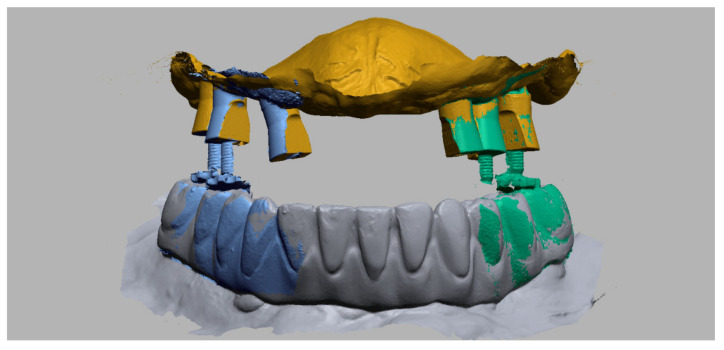
Same patient: A digital impression of the maxillary arch with dual-function scan bodies, a digital mandibular antagonist impression and two bilateral buccal bite-scans with adjusted bite pillars inserted into the screw holes of the dual-function scan bodies on multi-unit level (Geomagic Design X, 3D Systems Inc., Rock Hill, SC, USA).

Another advantage of the bite pillars is that patients do not have to bite in a moldable material. This decreases the risk for anterior displacement or fading from the hinge position. In particular, patients with strangulation reflex, restless patients or patients with a voluminous tongue will benefit from this approach (Figure 15 and Figure 16). Conversely, an irregular opposing dentition might prevent a stable support for the bite pillar or cause a deviation of the mandibular closure path. The clinician should pay attention to this and correct the contact if possible, which requires certain experience from the clinician. Similarly, sufficient bite pillars should be in contact with the opposing dentition, preferably in both quadrants of the arch, to provide stability. This might be a problem when several antagonistic teeth are missing. This technique might also be used for full mouth rehabilitation, when both arches are edentulous. In that case, however, opposing scan bodies and bite pillars need to be in contact or an additional bite registration paste should be used to fabricate an occlusal table. The latter, however, will hamper the registration procedure and reduce the precision.

## 5. Conclusions

The novel digital bite registration technique with dual-function scan bodies and bite pillars allows for a full-digital workflow for full-arch implant supported restorations. The digital bite workflow was 60% faster, and the overall deviation around 1 mm, which can be considered clinically acceptable.

## 6. Patents

This manuscript is related to United States patent application no. 16/339,394 published as 20190223990 for NUYTENS Philippe entitled “Scan post, bite pillar, reference pillar and related methods for recording dental implant position”.

## Figures and Tables

**Table 1:** Description and *p* values for all results.

	Mean (µm)	Standard Deviation (µm)	Minimum (µm)	Maximum (µm)	95.0% Lower CL for Mean (µm)	95.0% Upper CL for Mean (µm)	*p* Value
***Y*-axis**	200	744	−2.361	1.376	−348	−53	0.008
**Z-Axis**	67	752	−1.660	1.269	−82	216	0.374
***X*-axis**	46	731	−2.271	2.135	−99	191	0.529
**Absolute *Y*-axis**	611	470	10	2360	518	700	0.001
**Absolute Z-Axis**	630	410	10	1660	552	710	0.001
**Absolute *X*-axis**	470	560	10	2270	364	580	0.001
**Distance3D**	1115	668	150	3160	980	1246	<0.001

## Data Availability

Data are available from the corresponding author upon request.

## References

[1] Needles J.W. (1923). Mandibular Movements and Articulator Design. J. Am. Dent. Assoc..

[2] Niswonger M.E. (1938). Obtaining the Vertical Relation in Edentulous Cases That Existed Prior to Extraction. J. Am. Dent. Assoc..

[3] Zitzmann N.U., Marinello C.P. (1999). Treatment plan for restoring the edentulous maxilla with implant-supported restorations: Removable overdenture versus fixed partial denture design. J. Prosthet. Dent..

[4] Atwood D.A. (1966). A critique of research of the rest position of the mandible. J. Prosthet. Dent..

[5] Rugh J.D., Drago C.J. (1981). Vertical dimension: A study of clinical rest position and jaw muscle activity. J. Prosthet. Dent..

[6] Tryde G., Stoltze K., Fujii H., Brill N. (1977). Short-term changes in the perception of comfortable mandibular occlusal positions. J. Oral Rehabil..

[7] Niswonger M.E. (1934). The Rest Position of the Mandible and the Centric Relation. J. Am. Dent. Assoc..

[8] Wilson P.H.R., Banerjee A. (2004). Recording the retruded contact position: A review of clinical techniques. Br. Dent. J..

[9] Posselt U. (1952). Studies in the mobility of the human mandible. Acta Odontol. Scand..

[10] Ingervall B. (1964). Retruded contact position of the mandible. A comparison between children and adults. Odont. Revy.

[11] Kabcenell J.L. (1964). Effect of clinical procedures on mandibular position. J. Prosthet. Dent..

[12] Hellsing G., McWilliam J.S. (1985). Repeatability of the mandibular retruded position. J. Oral Rehabil..

[13] Schuyler C.H. (1969). Freedom in centric. Dent. Clin. North Am..

[14] Abbo B., Razzoog M.E. (2007). Transferring the existing occlusal vertical dimension using a duplicate denture. J. Prosthet. Dent..

[15] Clark W.A., Duqum I., Kowalski B.J. (2019). The digitally replicated denture technique: A case report. J. Esthet. Restor. Dent..

[16] Michalakis K.X., Touloumi F., Calvani L., Bedi A., Hirayama H. (2011). Simplifying Prosthetic Procedures while Converting an Interim Maxillary Removable Complete Denture to an Interim Implant-Supported Fixed Complete Denture. J. Prosthodont..

[17] Parnia F., Moslehifard E., Motayagheni N., Pournasrollah A. (2014). A time-saving method for transferring occlusal vertical dimension and centric relation of complete denture to a full-arch implant prosthesis. J. Contemp. Dent. Pract..

[18] Park D.H., Park J.M., Choi J.W., Kang E.S. (2017). Accuracy of several implant bite registration techniques: An in-vitro pilot study. J. Adv. Prosthodont..

[19] Aprile H., Saizar P. (1947). Gothic Arch Tracing and Temporomandibular Anatomy. J. Am. Dent. Assoc..

[20] Thakur M., Jain V., Parkash H., Kumar P. (2012). A Comparative Evaluation of Static and Functional Methods for Recording Centric Relation and Condylar Guidance: A Clinical Study. J. Indian Prosthodont. Soc..

[21] Raigrodski A.J., Sadan A., Carruth P.L. (1998). A Technique to Stabilize Record Bases for Gothic Arch Tracings in Patients with Implant-Retained Complete Dentures. J. Prosthodont..

[22] Joda T., Ferrari M., Gallucci G.O., Wittneben J.G., Bragger U. (2000). Digital technology in fixed implant prosthodontics. Periodontology.

[23] Joda T., Brägger U. (2014). Complete digital workflow for the production of implant-supported single-unit monolithic crowns. Clin. Oral Implants Res..

[24] Lepidi L., Galli M., Mastrangelo F., Venezia P., Joda T., Wang H., Li J. (2021). Virtual Articulators and Virtual Mounting Procedures: Where Do We Stand?. J. Prosthodont. Dent..

[25] Lepidi L., Chen Z., Ravida A., Lan T., Wang H.L., Li J. (2019). A Full-Digital Technique to Mount a Maxillary Arch Scan on a Virtual Articulator. J. Prosthodont..

[26] Lepidi L., Suriano C., Wang H.L., Granata S., Joda T., Li J. (2022). Digital fixed complete-arch rehabilitation: From virtual articulator mounting to clinical delivery. J. Prosthet. Dent..

[27] Hong S.J., Choi Y., Park M., Paek J., Pae A., Kim H.S., Kwon K.R., Noh K. (2020). Setting the Sagittal Condylar Inclination on a Virtual Articulator Using Intraoral Scan of Protrusive Interocclusal Position and Cone Beam Computed Tomography. J. Prosthodont..

[28] Griseto N.T., Gallucci G.O. (2021). Digital maxillomandibular relationship registration for an edentulous maxilla: A dental technique. J. Prosthet. Dent..

[29] Monaco C., Ragazzini N., Scheda L., Evangelisti E. (2018). A fully digital approach to replicate functional and aesthetic parameters in implant-supported full-arch rehabilitation. J. Prosthodont. Res..

[30] An X., Fang J.H., Jeong S.M., Choi B.H. (2018). A CAD-CAM technique for conversion of interim-to-definitive restoration in patients with complete edentulism. J. Prosthet. Dent..

[31] Michelinakis G., Nikolidakis D. (2019). Using the surgical guide for impression-free digital bite registration in the edentulous maxilla—a technical note. Int. J. Implant. Dent..

[32] Ahmed W.M., Verhaeghe T.V., Mccullagh A. (2021). Maxillary complete-arch implant-supported restoration: A digital scanning and hence- mandibular relationship workflow. J. Prosthet. Dent..

[33] Shao J., Qing H., Zhu Z., Li L. (2019). CAD-CAM–fabricated interim fixed complete-arch implant-supported restorations based on the existing dentition. J. Prosthet. Dent..

[34] Papaspyridakos P., Chen Y.W., Gonzalez-Gusmao I., Att W. (2019). Complete digital workflow in prosthesis prototype manufacture for complete-arch implant rehabilitation: A technique. J. Prosthet. Dent..

[35] Espona J., Roig E., Ali A., Roig M. (2020). Immediately loaded interim complete-arch implant-supported fixed dental prostheses fabricated with a completely digital workflow: A clinical technique. J. Prosthet. Dent..

[36] Kim J.E., Amelya A., Shin Y., Shim J.S. (2017). Accuracy of intraoral digital impressions using an artificial landmark. J. Prosthet. Dent..

[37] Cappare P., Sannino G., Minoli M., Montemezzi P., Ferrini F. (2019). Conventional versus Digital Impressions for Full Arch Screw-Retained Maxillary Rehabilitations: A Randomized Clinical Trial. Int. J. Environ. Res. Public Health..

[38] Rutkunas V., Gedrimiene A., Akulauskas M., Fehmer V., Sailer I., Jegelevicius D. (2021). In vitro and in vivo accuracy of full-arch digital implant impressions. Clin. Oral Implants Res..

[39] Schimmel M., Akino N., Srinivasan M., Wittneben J.G., Yilmaz B., Abou-Ayash S. (2021). Accuracy of intraoral scanning in completely and partially edentulous maxillary and mandibular jaws: An in vitro analysis. Clin. Oral Investig..

[40] Albayrak B., Sukotjo C., Wee A.G., Korkmaz İ.H., Bayındır F. (2021). Three-Dimensional Accuracy of Conventional Versus Digital Complete Arch Implant Impressions. J. Prosthodont..

[41] Knechtle N., Wiedemeier D., Mehl A., Ender A. (2021). Accuracy of digital complete-arch, multi-implant scans made in the edentulous jaw with gingival movement simulation: An in vitro study. J. Prosthet. Dent..

[42] Baghani M.T., Shayegh S.S., Johnston W.M., Shidfar S., Hakimaneh S.M.R. (2021). In vitro evaluation of the accuracy and precision of intraoral and extraoral complete-arch scans. J. Prosthet. Dent..

[43] Pesce P., Pera F., Setti P., Menini M. (2018). Precision and Accuracy of a Digital Impression Scanner in Full-Arch Implant Rehabilitation. Int. J. Prosthodont..

[44] Wong K.Y., Esguerra R.J., Chia V.A.P., Tan Y.H., Tan K.B.C. (2018). Three-Dimensional Accuracy of Digital Static Interocclusal Registration by Three Intraoral Scanner Systems. J. Prosthodont..

[45] D’haese R., Vrombaut T., Roeykens H., Vandeweghe S. (2021). In Vitro Accuracy of Digital and Conventional Impressions for Full-Arch Implant-Supported Prostheses. J. Clin. Med..

[46] Abdulateef S., Edher F., Hannam A.G., Tobias D.L., Wyatt C.C.L. (2020). Clinical accuracy and reproducibility of virtual interocclusal records. J. Prosthet. Dent..

[47] Zimmermann M., Ender A., Attin T., Mehl A. (2018). Accuracy of Buccal Scan Procedures for the Registration of Habitual Intercuspation. Oper Dent..

[48] Solaberrieta E., Otegi J.R., Goicoechea N., Brizuela A., Pradies G. (2015). Comparison of a conventional and virtual occlusal record. J. Prosthet. Dent..

[49] Camcı H., Salmanpour F. (2021). A new technique for testing accuracy and sensitivity of digital bite registration: A prospective comparative study. Int. Orthod..

[50] Kattadiyil M.T., Alzaid A.A., Campbell S.D. (2021). What Materials and Reproducible Techniques May Be Used in Recording Centric Relation? Best Evidence Consensus Statement. J. Prosthodont..

[51] Ren S., Morton D., Lin W.S. (2020). Accuracy of virtual interocclusal records for partially edentulous patients. J. Prosthet. Dent..

[52] Eriksson A., Ockert-Eriksson G., Lockowandt P., Eriksson O. (2002). Clinical factors and clinical variation influencing the reproducibility of interocclusal recording methods. Br. Dent. J..

[53] Edher F., Hannam A.G., Tobias D.L., Wyatt C.C.L. (2018). The accuracy of virtual interocclusal registration during intraoral scanning. J. Prosthet. Dent..

[54] Gintaute A., Keeling A.J., Osnes C.A., Zitzmann N.U., Ferrari M., Joda T. (2020). Precision of maxillo-mandibular registration with intraoral scanners in vitro. J. Prosthodont. Res..

[55] Resende C., Barbosa T., Moura G.F., Tavares L., Rizzante F., George F.M., Neves F., Mendonça G. (2021). Influence of operator experience, scanner type, and scan size on 3D scans. J. Prosthet. Dent..

[56] Róth I., Czigola A., Joós-Kovács G.L., Dalos M., Hermann P., Borbély J. (2020). Learning curve of digital intraoral scanning—An in vivo study. BMC Oral Health.

[57] Al Hamad K.Q. (2020). Learning curve of intraoral scanning by prosthodontic residents. J. Prosthet. Dent..

[58] Michelinakis G., Apostolakis D., Kamposiora P., Papavasiliou G., Özcan M. (2021). The direct digital workflow in fixed implant prosthodontics: A narrative review. BMC Oral Health.

[59] Gimenez-Gonzalez B., Hassan B., Özcan M., Pradíes G. (2017). An In Vitro Study of Factors Influencing the Performance of Digital Intraoral Impressions Operating on Active Wavefront Sampling Technology with Multiple Implants in the Edentulous Maxilla. J. Prosthodont..

[60] Rutkūnas V., Gečiauskaitė A., Jegelevičius D., Vaitiekūnas M. (2017). Accuracy of digital implant impressions with intraoral scanners. A systematic review. Eur. J. Oral Implantol..

[61] Nuytens P. (2017). Scan Post, Bite Pillar, Reference Pillar and Related Methods for Recording Dental Implant Position. US Patent.

